# Expression Sensitivity Analysis of Human Disease Related Genes

**DOI:** 10.1155/2013/637424

**Published:** 2013-11-24

**Authors:** Liang-Xiao Ma, Ya-Jun Wang, Jing-Fang Wang, Xuan Li, Pei Hao

**Affiliations:** ^1^Shanghai Center for Bioinformation Technology, Shanghai 201203, China; ^2^Key Laboratory of Synthetic Biology, Shanghai Institutes for Biological Sciences, Chinese Academy of Sciences, Shanghai 200031, China; ^3^Key Laboratory of Systems Biomedicine (Ministry of Education), Shanghai Jiao Tong University, Shanghai 200240, China; ^4^Pathogen Diagnostic Center, Institute Pasteur of Shanghai, Chinese Academy of Sciences, Shanghai 200031, China

## Abstract

*Background*. Genome-wide association studies (GWAS) have shown its revolutionary power in seeking the influenced loci on complex diseases genetically. Thousands of replicated loci for common traits are helpful in diseases risk assessment. However it is still difficult to elucidate the variations in these loci that directly cause susceptibility to diseases by disrupting the expression or function of a protein currently. *Results*. We evaluate the expression features of disease related genes and find that different diseases related genes show different expression perturbation sensitivities in various conditions. It is worth noting that the expression of some robust disease-genes doesn't show significant change in their corresponding diseases, these genes might be easily ignored in the expression profile analysis. *Conclusion*. Gene ontology enrichment analysis indicates that robust disease-genes execute essential function in comparison with sensitive disease-genes. The diseases associated with robust genes seem to be relatively lethal like cancer and aging. On the other hand, the diseases associated with sensitive genes are apparently nonlethal like psych and chemical dependency diseases.

## 1. Introduction

To elucidate the etiology and pathogenesis of the diseases, scientists have made efforts to map human disease loci genetically and clone many diseases genes [1, 2]. Recently with the development of new sequencing technology and high throughput microarray technology, the searching for the genetic traits of the diseases and scanning of personal genomic variations are revolutionized. Genome-wide association studies (GWAS) [3] showed its strong abilities in detecting complicated genetic variations in genes and building genomic variation patterns compared with linkage analysis [4] and candidate gene studies [5]. GWAS have made contribution to establish plenty of disorder-gene association pairs [6]. As reported, over 4008 SNPs are associated with 819 common diseases [7]. The Genetic Association Database (GAD) [8] is a valuable resource of human genetic association studies on complex diseases and disorders, which facility us to rapidly establish the relationship of disorder-gene association pairs. The association studies explained the relationships between the diseases and genes on the genomic levels. Diseases-associated studies have identified functional genetic variations, but they didn't well make sure the variations could cause the diseases directly. Annotating the diseases associated variations on different levels are necessary to identify the outstanding risking genes. Here we wonder whether the genes associated the same diseases show similar expression features? 

In order to probe the expression feature of disease genes which are detected by the associated studies, we referred to the method of gene expression sensitivity analysis [9]. We firstly investigated human global gene expression characters in response to the environmental perturbation. Gene expression patterns are different in various biological conditions, a lot of case-control expression patterns have been profiled using the high throughput microarray technology. Studies show a group of genes' expression that could be easily disturbed with various external stimulations [9, 10]; however, some genes are stably expressed in different environments, which indicate that the genes show different expression sensitivity. For example, housekeeping genes which have been well investigated in maintaining the basal cellular functions have revealed its expression stability [11, 12]. Recently gene expression sensitivity to external stimulation have been studied in yeast and human [9, 10], which guide us to generate the idea to investigate the expression sensitivity of diseases genes.

It is worthy to obtain a global view of the intrinsic properties of human disease gene expression as a response to perturbations. Gene Expression Omnibus (GEO) database [13] and Genetic Association Database (GAD) [8] were used to analyze the human associated gene expression sensitivity. A meta-analysis method could be used to seek the sensitive genes and robust genes in the expression profiles globally. Based on our calculation of sensitive values of gene expression, we firstly categorized the genes into robust and sensitive groups. Furthermore we investigated the expression sensitivity of disease related genes in response to the perturbations and found some of genes were detected by the association studies previously, but the expression of these genes is relatively stable in their corresponding disease studies. The results also indicate some diseases related genes that show their expression robustness (DGR) like cancer and aging genes, and chemical dependency disease related genes seem to be relatively sensitive (DGS).

## 2. Materials and Computational Methods

### 2.1. Data Collection and Preprocessing

 The Genetic Association Database (GAD) includes over 80,000 gene records of genetic association studies. Importantly, the database has a designation of whether the gene record was reported to be associated with disease phenotype. The option Y means that the gene of record was associated with the disease phenotype; otherwise, the option N was not associated. We collected the records in GAD that associated with the disease phenotype and got the records only annotated with the standard disease phenotype keywords from MeSH (http://www.nlm.nih.gov/mesh/) vocabulary. 

After filtering, 13277 records were used for further investigation. In our study, 2588 disease related genes were acquired from 13275 records from the Genetic Association Database. Among these genes, 1804 genes were associated with more than one disease and 784 were associated with only one disease (Supplementary Material available online at http://dx.doi.org/10.1155/2013/637424, Table S2). These diseases related genes are mainly from human genetic association studies of complex diseases and disorders. 1464 kinds of diseases were extracted from 13275 records. To establish the relationships between genes and their corresponding diseases groups, the 1464 kinds of diseases were divided into 16 groups by paring database. 

We downloaded the HGU133plus2.0 microarray datasets from the GEO database, each dataset had been normalized with MAS5 when the authors submitted them into the database as required (http://www.ncbi.nlm.nih.gov/geo/). To calculate the expression level of each gene, we referred to the methods from the previous work [9]. We discarded the data sets with less than 6 arrays and changed the expression values into 10 if the expression values are less than 10. Then the expression values of all probes were logarithmic transformed (base 2). We choose the maximum expression value as gene expression value if multiple probes illustrate the same gene expression.

### 2.2. Calculate Sensitive Values (SV) of Each Gene

In our study, 167 datasets (labeled as *M* in the Formula below) in GEO were used to calculate the sensitive values of genes. In each dataset *j*, we calculate the standard deviation (SD) and mean of each gene (*g*_*i*_), getting the coefficient of variation (CV) of each gene (*g*_*i*_) with SD divided mean. The CV of each gene in each dataset is calculated as follows:

(1) CVgij=SD(gij)mean(gij).

In order to merge the results of different datasets and minimum experimental variation during sensitivity analysis, we employed the large scale meta-analysis method reported in our previous work [9]. We ranked the CV of genes in each dataset and constructed a matrix of ranked CV to datasets. Sensitive values (SV) are calculated as mean of each ranked CVs of all datasets:

(2) SVgi=∑j=1Mrank⁡(CVgij)M.

### 2.3. Defining Sensitive Genes and Robust Genes

In the current study, we tried to establish the relationships between the different kinds of disease and different sensitive genes. Firstly we selected a group of genes whose expression could be significantly disturbed and a group of genes whose expression significantly stable. After calculating the sensitive values of genes, we used the 5 percent as the cutoff value. The top 5 percent of genes are significantly sensitively expressed, we took five percent of genes with lowest sensitive values as robust genes groups, and five percent of genes with highest SV were considered as sensitive genes.

## 3. Results

**Figure 1:**
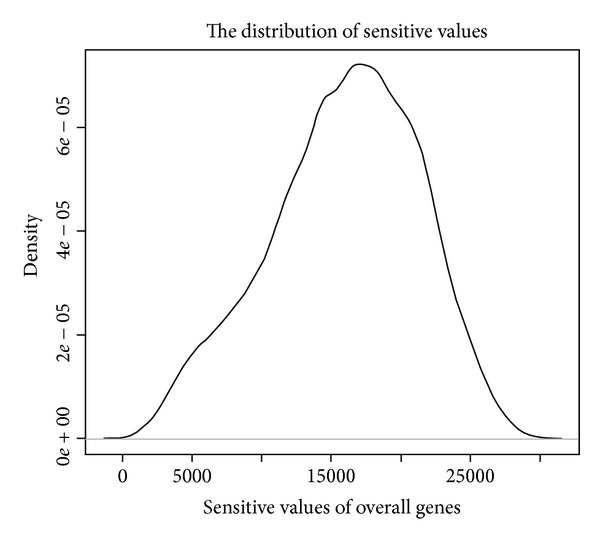
This figure demonstrates the distribution of the average rank order of gene expression standard deviations. The distribution of SV is skewed normal distribution.

The Affymetrix HGU 133a plus 2.0 microarray covers 31835 genes, which represents more genes than the HGU133a microarray does. The distribution of SV is skewed normal distribution (Figure 1). The figure suggested that there are more genes with a comparatively lower sensitive value than those with higher sensitive values indicating existing more robust genes than sensitive genes. Although the distribution of sensitive values are skewed normal, we took five percent of genes with lowest sensitive values as robust genes groups, and also five percent of genes with highest SV were considered as sensitive genes. Therefore, we got 1592 robust genes and sensitive genes individually. Therefore, we got 1592 robust genes and 1592 sensitive genes. After comparison with disease related genes, we got 131 diseases related robust genes and 467 disease related sensitive genes respectively (Supplementary Table S1).

### 3.1. Functional Annotations of Expression Robust and Sensitive Genes

**Figure 2:**
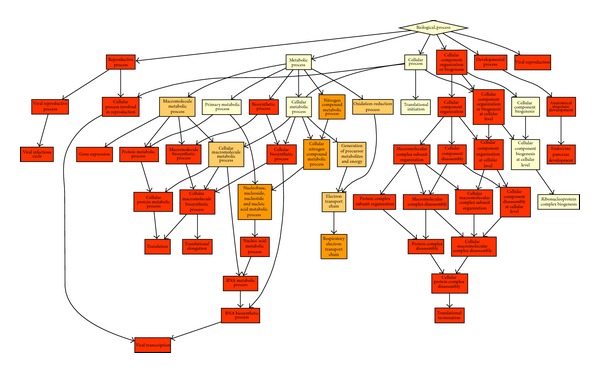
The biological process GO enrichment graph illustrates that the robust genes play basic roles of cell developments. The color in the rectangle is close to red; the genes are more enriched in that GO module, and the white color indicates the least enrichment in that module.

**Figure 3:**
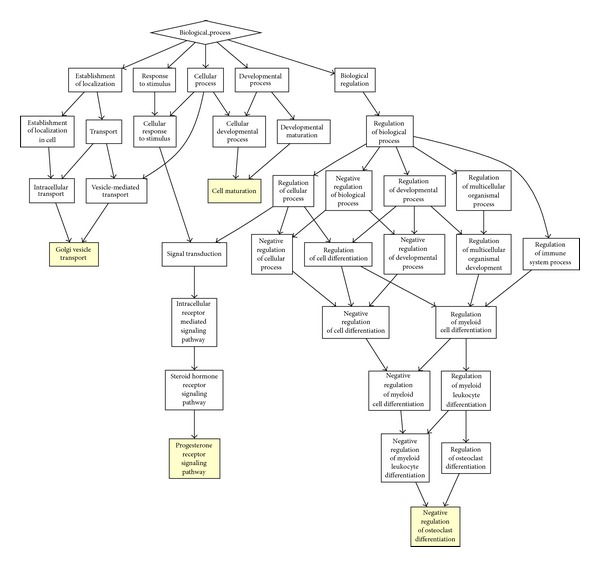
The biological process GO enrichment graph illustrates that the sensitive genes mainly regulate the cell metabolism. The sensitive genes are enriched in the GO module with a yellow color.

In order to identify the biological functions in the cell, we conducted GO enrichment analysis [14, 15]. The results show that robust genes are strongly engaged in the cellular component organization (GO: 0071842), reproductive process (GO: 0022414), and viral reproduction (GO: 0016032) (Figure 2 and Table 1). However the sensitive genes play important roles in progesterone receptor signaling pathway (GO: 0050847) and negative regulation of osteoclast differentiation (GO: 0045671) (Figure 3 and Table 1). Furthermore enrichments analysis for cell components modules indicates that the robust gene are strongly enriched in the intracellular (GO: 0005622) and ribosome (GO: 0005840) (Figure 3), but the sensitive genes didn't show enrichment in the cell components. Based on the above observation, we conclude that the robust genes are engaged in the basic biological process.

### 3.2. A Case about Robust Genes Consistently Expressing in Their Corresponding Disease

We have found the expression of robust genes are stable in various conditions and inferred that the some disease related robust genes may express consistently in their corresponding diseases conditions. To verify our assumption, we took colorectal cancer and its associated robust genes HIF1A and MLH as an example. 

**Figure 4:**
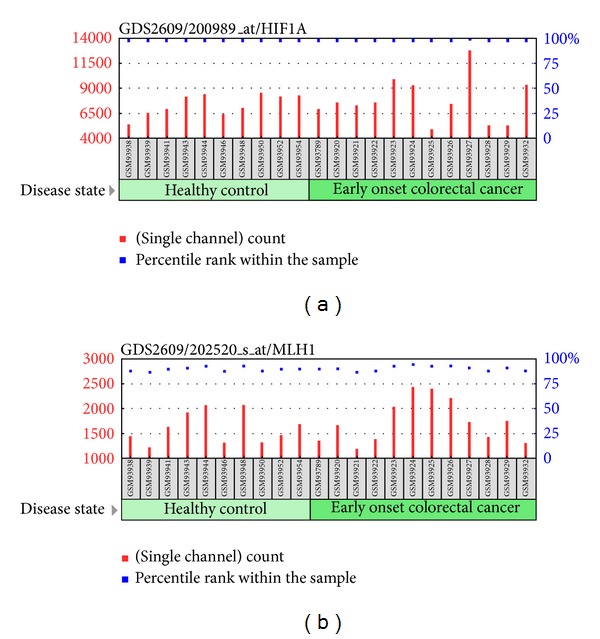
(a) The expression profile of gene HIF1A in GDS2609. (b) The expression profile of MLH1 in GDS2609.

HIF1A associated with colorectal cancer is one of robust genes. Hypoxia-inducible factor-1 (HIF1) is a heterodimer composed with HIF1A and HIF1B. HIF1 is functionally important in cellular and systemic homeostatic responses to hypoxia and initially found as transcription factor in mammalian cells cultured under reduced oxygen tension [16]. Fransén et al. found that polymorphic alleles in the gene of HIF1A show significant higher risk for the development of ulcerative colorectal cancer, which indicates that the HIF1A polymorphisms display their importance in the development of ulcerative intestinal tumors [17]. To view the gene expression variations of colorectal cancer, we took a case from a work [18] that analyzed expression changes in early onset colorectal cancer (GDS2609). The expression of HIF1A didn't show significant change in the study (Figure 4(a)). The robust gene HIF1A might be ignored in the colorectal cancer expression analysis. 

Another robust gene MLH1 involved in DNA mismatch repair is also associated with colorectal cancer [19]. Liu et al. revealed that colorectal cancer is associated with 2 missense mutations in exon 16 of the MLH1 [20]. Chan et al. described a novel germline 1.8-kb deletion involving of the MLH1 gene associated with hereditary nonpolyposis colorectal cancer in a Hong Kong family [21]. Recently Nejda et al. suggests that gender should be considered in colorectal cancer association studies [22]. They found that nucleotide polymorphism in MLH1 displays a higher risk in sporadic colorectal carcinogenesis especially in men. A mechanism of genomic instability has been identified in colorectal cancer [23], the DNA mismatch repair genes MLH1 inactivated by hypermethylation of their promoter could cause microsatellite instability. We also found the expression of MLH1 in the early onset colorectal cancer investigation (GDS2609) is not significantly changed (Figure 4(b)). The expression profile analysis may overlook importance of robust gene MLH1. Therefore, we believe that diseases related robust genes are easily ignored in the expression analysis. In order to know the robust genes are enriched in what kinds of diseases, we established the relationships between diseases and gene expression sensitivities. 

### 3.3. The Relationships between Diseases and Gene Expression Sensitivity

 The 1469 diseases with their associated genes from GAD were divided into 16 groups. The disease genes are classified as robust disease gene groups and sensitive diseases genes groups based on whether they were included in the robust genes groups or sensitive genes groups. We performed the enrichment analysis of disease genes based on the hyper geometric distribution. The results (Table 2) show that the cancer, aging, and pharmacogenomics are enriched with robust genes (DGR), with *P* values of 0.001309, 0.025063, and 0.06734 individually, and psych, chemical dependency, and reproduction are enriched with sensitive genes (DGS), with corresponding *P* values of 0.001954, 0.028318, and 0.055457. 

**Figure 5:**
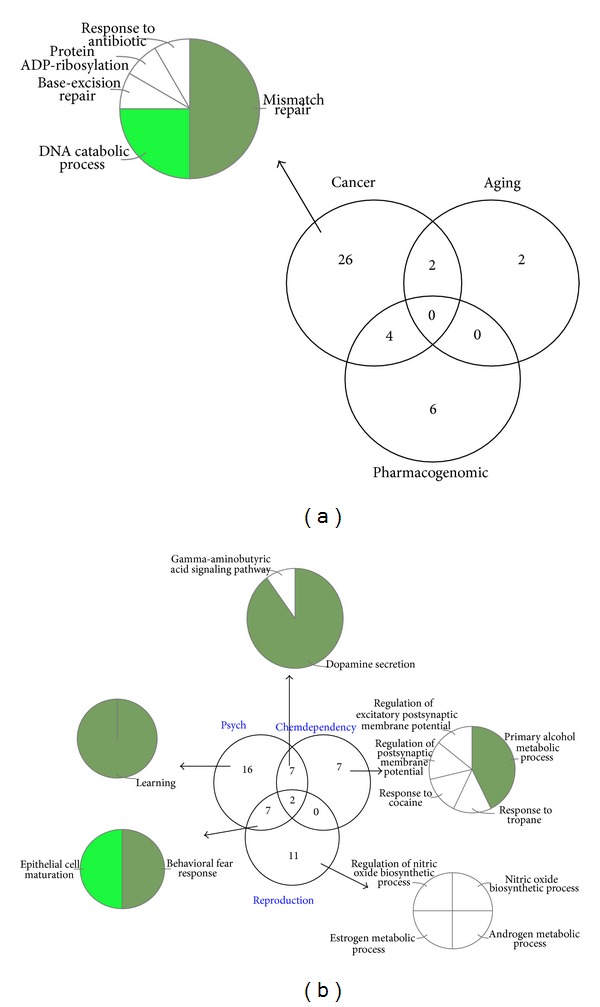
(a) Three kinds of disease genes show significant robustness. GO ontology analysis of cancer associated genes are on the left of graph. Cancer related robust genes apparently are more essential in the biological process. (b) Three kinds of diseases associated genes are relatively sensitive. The diseases associated with sensitive genes seem to be nonlethal.

We annotated the gene from DGR and DGS with Gene Ontology [24]. Firstly the DGR and GDS were classified into seven groups (Figure 5(a)) according to whether the genes associated one or more diseases. In the DGR groups, cancer genes are mainly engaged in the mismatch repair (GO: 0006298), DNA catabolic process (GO: 0006308), and base excision repair (GO: 0006284). The GO analysis of DGS (Figure 5(b)) shows the genes only associated with psych diseases are enriched in the learning (GO:0007612) process and the genes associated both psych and chemical dependency diseases are engaged in the dopamine secretion (GO:0014046) and gamma-aminobutyric acid signaling pathway (GO:0007214) and so forth. We conclude that the DGR mainly engaged in more essential biological process compared with DGS which mainly involve in the regulation and response process.

## 4. Discussion and Conclusion

We parsed the GAD databases and selected diseases associated genes. Because the human genes show different expression sensitivity in response to the environmental perturbation [9], we evaluated the expression features of diseases genes with the method of gene expression sensitivity analysis. Because the finding of expression of robust genes is not easily changed in various biological conditions, we assumed that the disease related robust genes might be expressed stably in their corresponding disease conditions. The colorectal cancer associated robust genes HIF1A and MLH did not show significant expression changes in the studies of colorectal cancer [17, 18, 20, 22]. Importantly our results suggested the genes associated with different diseases also reveal different sensitivities. We found the cancer, aging, and pharmacogenomics related genes display expression robustness, and psych, chemical dependency, and reproduction-associated genes are relatively sensitive. In our study, the robust disease related genes were investigated not only by combining the gene ontology but also by grouped disease information. The defect of robust genes could cause more lethal diseases, such as cancer and aging diseases. Thus diseases related robust genes might play more essential roles to keep health for human.

Additionally, the protein interaction network and gene ontology provide extensive information to detail the relationships between different diseases genes. It was found that the structure of a cellar network and its functional properties were connected with protein or “Hubs” which are more likely encoded by essential genes [25, 26]. Human robust genes are higher degree centrality than the random groups of genes in the protein interaction network [9]. Gene ontology analysis also indicates the robust genes play an essential role in the cellar biology. The robust genes have shown its importance in different levels. Recent studies reveal that some kinds of diseases genes potentially encode hubs [27, 28]. Goh et al. suggested that cancer genes are more likely to encode hubs in the human disease networks and show higher coexpression with the rest of the genes in the cell [29], which means that cancer genes play critical roles in cellar development and growth. Age-related diseases tend to attack the center of the human protein network [30]. PPI network investigation above indicated that the cancer and aging related genes are potentially robust.

Based on the studies above, we believe that the different diseases genes reveal distinct expression sensitivity. Diseases that are associated with robust genes seem to be lethal, and the diseases associated with sensitive genes are nonlethal apparently. The gene ontology analysis indicates the robust genes are more essential when compared with sensitive genes. The robust genes those stably express in various environmental conditions are easily ignored in the expression analysis. Therefore the consideration of sensitivity of disease genes might be greatly helpful in elucidating of etiology and pathogenesis of the diseases. In practice, calculation of the diseases genes' sensitive values could be used to predict the potential harm to heath. In addition, if a robust gene is a potential drug target, it would have little therapeutic effects to these diseases by disturbing the expression level of the robust genes.

## Supplementary Material

Table S1. The number of genes associated with 16 kinds of grouped diseases related genes.
Table S2. The number of diseases associated with each gene involved in the current study.

## Figures and Tables

**Table 1:** Enriched biological process. The table shows the main biological process that the robust genes and sensitive genes engaged. Most robust genes take part in the translational elongation and viral transcription; however, the sensitive genes prefer to respond to the progesterone receptor stimulation and regulation.

Robust genes		Sensitive genes	
Enriched biological process	*P* value	Enriched biological process	*P* value
Translational elongation	3.12*E* − 41	Progesterone receptor signaling pathway	1.74*E* − 4
Viral transcription	5.42*E* − 39	Negative regulation of osteoclast differentiation	7.11*E* − 4
Translational termination	8.26*E* − 38	Cell maturation	7.14*E* − 4
Protein complex disassembly	8.26*E* − 38	Golgi vesicle transport	9.89*E* − 4

**Table 2:** Expression sensitivity analysis of diseases genes. The cancer, aging, and pharmacogenomic related genes reveal their robustness, and psych chemdependency and reproduction associated genes show their sensitiveness.

Disease	Robustness	Sensitiveness	Disease	Sensitiveness	Robustness
Cancer	1.31*E* − 03	7.08*E* − 01	Psych	1.95*E* − 03	8.09*E* − 01
Aging	2.51*E* − 02	4.87*E* − 01	Chemdependency	2.83*E* − 02	4.32*E* − 01
Pharmacogenomic	6.73*E* − 02	9.44*E* − 01	Reproduction	5.55*E* − 02	1.85*E* − 01
