# Supplementary material for: Expression Sensitivity Analysis of Human Disease Related Genes
Source: Biomed Res Int. 2013 Nov 24;2013:637424. doi: 10.1155/2013/637424 (PMC3857905; doi:10.1155/2013/637424)
Supplement: Supplementary file 1 — Table S1. The number of genes associated with 16 kinds of grouped diseases related genes. Table S2. The number of diseases associated with each gene involved in the current study. [file 637424.f1.pdf]

Table 1 – The number of genes associated with 16 kinds of grouped diseases related genes

Diseases	Number of Disease related genes	Number of Disease related robust genes	Number of Disease related sensitive genes
**Aging**	33	3	3
**Cancer**	517	29	52
**Cardiovscular**	566	15	67
**Chemdependency**	97	3	16
**Developmental**	95	1	12
**Hematological**	149	3	19
**Immune**	635	16	68
**Infection**	190	4	25
**Metabolic**	651	20	75
**Neurological**	364	13	42
**Normalvariation**	13	0	0
**Pharmacogenomics**	149	8	10
**Psych**	260	6	42
**Renal**	116	3	15
**Reproduction**	136	6	20
**Vision**	74	1	10

Table 2 – The number of diseases associated with each gene

Genes	Number of related diseases	Genes	Number of related diseases	Genes	Number of related diseases
HLA-DRB1	136	TG	4	SOD1	2
TNF	135	TGFA	4	SOX10	2
NOS3	126	THADA	4	SPR	2
HLA-DQB1	120	THSD1	4	SPRY2	2
ACE	112	TIMP1	4	SPRY4	2
Intergenic	109	TIRAP	4	SRD5A1	2
MTHFR	99	TLR3	4	SREBF2	2
IL10	87	TLR6	4	SSTR2	2
TP53	87	TMPRSS6	4	ST8SIA2	2
APOE	86	TNFA	4	STG	2
IFNG	78	TNFSF10	4	STK33	2
SERPINE1	78	TNFSF15	4	STMN2	2
IL6	76	TNNT2	4	SULT1A2	2
IL1B	75	TPM1	4	SULT1E1	2
VDR	71	TTC12	4	SULT2A1	2
CTLA4	70	TUBB	4	SURF1	2
CD14	69	TXNIP	4	SYNJ1	2
PON1	66	UCHL1	4	SYP	2
HLA-DQA1	63	ZFHX3	4	TACR1	2
TLR4	63	ZNRD1	4	TACR2	2
SLC6A4	62	ABCA4	3	TAF5L	2
VEGFA	61	ABCC8	3	TAGAP	2
TGFB1	58	ABCG5	3	TBX22	2
GSTM1	57	ABL1	3	TCF7	2
IL1RN	56	ACSL6	3	TCS2	2
MMP9	53	ACVRL1	3	TECTA	2
HLA	51	ADRA2C	3	THPO	2
CYP1A1	46	AHRR	3	THRA	2
GSTT1	45	AIM1	3	TIMD4	2
HLA-A	45	AIPL1	3	TMPRSS3	2
HLA-B	45	AKAP13	3	TNC	2
IL4	45	AKR1B10	3	TNFR2	2
MMP1	45	AKT1	3	TNFRSF13B	2
IL8	44	ALDH1A2	3	TNFRSF17	2
ABCB1	43	ALDOB	3	TNFRSF4	2
F5	43	ANGPT2	3	TNIP1	2
FCGR2A	43	ANGPTL3	3	TNPO3	2

CDKN2A	42	ANKS6	3	TNRC9	2
MMP3	42	APBB1	3	TOMM40	2
ESR1	41	APOC4	3	TOPBP1	2
MPO	41	APOD	3	TOR1B	2
AGT	40	APOH	3	TP53BP2	2
AR	40	APP	3	TRAF1-C5	2
NAT2	40	ARL6	3	TRAF5	2
SOD2	40	ARSA	3	TRIM10	2
CCND1	39	ARSB	3	TRIM38	2
ITGB3	39	ASIP	3	TRNAA-UGC	2
COMT	38	ASPN	3	TRPM2	2
CRP	38	ATP13A2	3	TSC22	2
CYP17A1	38	ATP2A1	3	TSLP	2
LTA	38	ATP8B1	3	TSPAN7	2
BDNF	37	ATR	3	TSR1	2
F2	37	ATXN2	3	TTC39B	2
PPARG	37	BAK1	3	UBQLN1	2
IL1A	36	BAX	3	UGT2B15	2
NOS2A	36	BCDIN3D	3	UNC13B	2
MAOA	35	BCL10	3	UQCRFS1	2
ADRB2	34	BGLAP	3	UTS2R	2
CCL2	33	BLK	3	VAMP1	2
CCR5	33	BLMH	3	VAMP8	2
LPL	33	BMP6	3	VARS2	2
SELE	33	BPI	3	VAX1	2
DRD2	32	BRD2	3	WDR1	2
DRD4	32	BTC	3	WDR36	2
FAS	32	C1GALT1	3	WNK1	2
GSTP1	32	C2orf47	3	WTAP	2
APOB	31	C6orf10	3	WWOX	2
CCR2	31	CACNG3	3	XKR6	2
HMOX1	31	CALD1	3	ZBTB38	2
MBL2	31	CAST	3	ZBTB40	2
NQO1	31	CCL24	3	ZC3H7A	2
PTPN22	31	CD226	3	ZDHHC8	2
ADIPOQ	30	CD38	3	ZNF366	2
CYP2D6	30	CD4	3	ZNF429	2
CYP2E1	30	CDC2	3	ZNF699	2
FGB	30	CDC6	3	ZNF74	2
GNB3	30	CDH2	3	A2BP1	1
HTR2A	30	CDKN1C	3	AAK1	1
LIPC	30	CETN3	3	AB002360	1
APOA1	29	CHGA	3	AB116074	1
ERCC2	29	CHIT1	3	ABCA12	1

IL18	29	CHM	3	ABCC9	1
PTGS2	29	CHRM2	3	ABP1	1
UGT1A1	29	CHRNB2	3	AC020743.7	1
XRCC1	29	CHRNB4	3	AC026790.5	1
HP	28	CHST8	3	AC104108.3	1
IL4R	28	CILP2	3	ACADM	1
TNFRSF1A	28	CMYA5	3	ACAT2	1
APOC3	27	CNR2	3	ACOXL	1
COL1A1	27	COL4A3	3	ACP5	1
DRD3	27	COL5A1	3	ACSM1	1
HFE	27	COL6A1	3	ACTG1	1
IL13	27	COL9A2	3	ACTN3	1
ITGA2	27	CPVL	3	ACVR2B	1
NPY	27	CRB1	3	ADAM19	1
CYP1B1	26	CSF3	3	ADAM30	1
HLA-DPB1	26	CTNNB1	3	ADAMTS9	1
KRAS	26	CTSB	3	ADCY5	1
MIF	26	CX3CL1	3	ADD3	1
EGFR	25	CYP21A1P	3	ADH7	1
IL1R1	25	DAO	3	ADRA1D	1
APOA5	24	DARC	3	AGL	1
CETP	24	DAZL	3	AHCY	1
F13A1	24	DEFB4	3	AICDA	1
FCGR3A	24	DGCR2	3	AK092739	1
ICAM1	24	DGKG	3	AK1	1
LDLR	24	DLX3	3	AK123267	1
MMP2	24	DNASE2	3	AK3	1
TLR2	24	DNMT3B	3	AKAP11	1
APC	23	DPF3	3	AKD2	1
CYP11B2	23	DRP2	3	AKR1C3	1
CYP19A1	23	DSG3	3	AL079307.7	1
CYP2C19	23	ECE1	3	AL157404.18	1
FASLG	23	ECG2	3	ALAD	1
IGF1	23	EFCAB4B	3	ALDH1A1	1
INS	23	EGR2	3	ALDH3A2	1
PON2	23	EN2	3	ALG10B	1
TAP2	23	ERAP2	3	AMPA 1	1
ADD1	22	ERCC3	3	ANKRD30A	1
ENPP1	22	ERCC4	3	ANP32B	1
GP1BA	22	ERDA1	3	ANXA5	1
IL2	22	ETV5	3	AOAH	1
KL	22	EXT2	3	APBB3	1
LEPR	22	F2R	3	APOCII	1
TPH1	22	FADS3	3	APOL1	1

AGTR1	21	FAIM2	3	AQP1	1
CCL5	21	FEZ1	3	ARF7	1
CX3CR1	21	FGG	3	ARHGAP1	1
CYP2C9	21	FLI1	3	ARHGEF12	1
CYP3A4	21	FRDA	3	ARID2	1
EPHX1	21	FRZB	3	ARID5B	1
MDM2	21	FYN	3	AS3MT	1
PPARA	21	GALNT2	3	ASAP1	1
PTPN11	21	GART	3	ATG5	1
UCP2	21	GAS1	3	ATP2A2	1
CFTR	20	GCGR	3	ATP5O	1
CYBA	20	GCLM	3	ATP6	1
ESR2	20	GCNT2	3	ATXN1	1
F8	20	GDF15	3	ATXN8OS	1
FABP2	20	GJA4	3	AVPR1A	1
HNF1A	20	GLI2	3	AVPR1B	1
IL12B	20	GPR1	3	B3GALT4	1
OGG1	20	GRIK2	3	B4GALT6	1
RAGE	20	GRM5	3	BACE1	1
TCF7L2	20	HCR	3	BACH2	1
XRCC3	20	HD	3	BC016328	1
AGER	19	HER2	3	BC042052	1
CARD15	19	HERC2	3	BCL2L11	1
EDN1	19	HK1	3	BE789145	1
IGF2	19	HNMT	3	BET1L	1
NOD2	19	HOMER1	3	BICD1	1
NOS1	19	HOXA1	3	BLID	1
SERPINA3	19	HPRT1	3	BLM	1
SLC6A3	19	HRC	3	BMPR2	1
TH	19	HSD11B2	3	BNC2	1
ALDH2	18	HTR5A	3	BOLL	1
CDH1	18	HTRA1	3	BPY2	1
CDKN2B	18	IBD2	3	BQ013595	1
CYP1A2	18	IDDM2	3	BRDG1	1
F7	18	IFIH1	3	BRUNOL4	1
HSPA1B	18	IFNA1	3	BST1	1
INSR	18	IFNA13	3	C10orf32	1
OPRM1	18	IFNA17	3	C12orf43	1
RET	18	IFNAR2	3	C12orf5	1
TNFRSF1B	18	IGHG1	3	C14orf118	1
UGT1A7	18	IL12RB2	3	C16orf47	1
WT1	18	IL1RA	3	C16orf75	1
ADRB3	17	IL21	3	C17orf69	1
BCL2	17	IL3	3	C1orf125	1

HLA-C	17	IRF2	3	C1QA	1
HNF4A	17	IRF6	3	C1QTNF6	1
IFNGR1	17	ITGAM	3	C20orf132	1
JAK2	17	ITGB2	3	C20orf133	1
MYCL1	17	ITIH3	3	C20orf46	1
PRNP	17	KCNJ10	3	C20orf82	1
RETN	17	3-Sep	3	C2CD4B	1
TAP1	17	KCNMB3	3	C2orf3	1
TCF1	17	KCNS3	3	C5	1
AHSG	16	KCTD15	3	C8orf35	1
APOA4	16	KIAA1598	3	C9orf72	1
CAT	16	KITLG	3	C9org82	1
CDKN1A	16	KLK2	3	CACNA1C	1
COL2A1	16	KLRC1	3	CACNA1H	1
FBN1	16	KLRC2	3	CAMK1D	1
GNAS	16	KLRD1	3	CAMK4	1
HIF1A	16	LAMB2	3	CAND1	1
IRS1	16	LAPTM4B	3	CANX	1
LEP	16	LBP	3	CARD9	1
MLH1	16	LGALS2	3	CASP7	1
NR3C1	16	LHB	3	CASQ1	1
PTPN1	16	LHCGR	3	CBLN2	1
VHL	16	LIF	3	CCDC26	1
WRN	16	LITAF	3	CCDC60	1
ABCA1	15	LMBRD2	3	CCL20	1
ADH1C	15	LOC64673	3	CCL21	1
APEX1	15	LOC72901	3	CCR6	1
BRCA2	15	LRCH1	3	CD164	1
CAPN10	15	LRMP	3	CD33	1
CHEK2	15	LRP8	3	CD58	1
FCGR3B	15	LRRC16A	3	CD6	1
FHIT	15	LRRC18	3	CD72	1
FTO	15	LSP1	3	CD80	1
HBB	15	LTB	3	CD81	1
MC1R	15	MAD1L1	3	CDC123	1
MGMT	15	MAFB	3	Cdc25A	1
MTR	15	MAML2	3	CDH12	1
NFKBIA	15	MAP3K1	3	CDH4	1
NPPA	15	MCCD1	3	CDK2	1
PLA2G7	15	MEF2A	3	CDK5	1
RAS	15	MEN1	3	CEBPE	1
SDHB	15	MMAB	3	CENPC1	1
STAT6	15	MPDZ	3	CENTD1	1
SULT1A1	15	MS	3	CERKL	1

ADH1B	14	MS4A1	3	CES1	1
ADRA2A	14	MSH6	3	CFB	1
ATM	14	MTHFD1L	3	CFL1	1
CDKN1B	14	MTNR1B	3	CHCHD9	1
ERBB2	14	MYH1	3	CHL1	1
F12	14	MYH7	3	CHRM5	1
GHR	14	NAMPT	3	CHRNA1	1
GHRL	14	NBN	3	chrna5 and chrna3	1
GPX1	14	NCR3	3	CHRND	1
HRAS	14	NDRG4	3	CHST6	1
IL23R	14	NEUROG3	3	CITED2	1
ITGA2B	14	NKX2-5	3	CKM	1
MICA	14	NPC1	3	CLCN1	1
MTRR	14	NPR1	3	CLCN2	1
NAT1	14	NR1I2	3	CLCNKB	1
PARP1	14	NSD1	3	CLDN14	1
PGR	14	NXPH2	3	CLGN	1
PLAU	14	OMP1	3	CNNM2	1
SLC19A1	14	OPRK1	3	CNTLN	1
THBD	14	ORMDL3	3	CNTN5	1
TLR9	14	P2RY2	3	CNTNAP5	1
ABCG2	13	PAFAH	3	COL4A5	1
BDKRB2	13	PAFAH1B1	3	COL9A1	1
CALCR	13	PBEF1	3	COL9A3	1
CASR	13	PCM1	3	CORIN	1
CDKAL1	13	PER3	3	CP	1
CFH	13	PHACTR1	3	CPAMD8	1
CMA1	13	PHF11	3	CPB2	1
CNR1	13	PHKB	3	CPT2	1
CXCL12	13	PICK1	3	CR603372	1
DBH	13	PIGA	3	CREB1	1
FGFR4	13	PLA2G2D	3	CRHR2	1
HLA-DRB5	13	PLA2G4A	3	CRIM1	1
IL6R	13	PLCL1	3	CRY2	1
KCNJ11	13	PLG	3	CRYGC	1
MC4R	13	PLTP	3	CSMD1	1
MTTP	13	POU6F2	3	CSNK1E	1
NDP	13	PPP1R3A	3	CSNK1G3	1
PPARGC1A	13	PPP2R2C	3	CST5	1
PTCH	13	PRDM5	3	CTBP2	1
SPP1	13	PRKAA2	3	CTCF	1
TP73	13	PROS1	3	CTNS	1
TYMS	13	PSMA6	3	CTSH	1
XPA	13	PTGER4	3	CUL5	1

XPC	13	PTPRF	3	CXCR4	1
AMPD1	12	PYY	3	CYP17	1
BARD1	12	RANKL	3	CYP21	1
BRCA1	12	RASAL2	3	CYP3A43	1
CD40	12	RDH5	3	CYP3A7	1
CR1	12	RDS	3	CYP4V2	1
CYP2A6	12	RGS2	3	CYTB	1
DRD5	12	RNASEL	3	DAAM2	1
EGF	12	RNF207	3	DAG1	1
G6PD	12	RPGR	3	DAP3	1
GCKR	12	RPS6KA3	3	DBC1	1
HSPA2	12	RXRB	3	DCAMKL1	1
MEFV	12	SAH	3	DCD	1
MHC	12	SALL4	3	DCDC2	1
NFKB1	12	SCA1	3	DCDC5	1
PLAT	12	SCGN	3	DDEF1	1
PPARD	12	SCL2A1	3	DDEF2	1
SCN5A	12	SDF1	3	DENND1B	1
SELP	12	SEC16B	3	DGAT1	1
SERPINA1	12	SERPINB2	3	DGKQ	1
SFTPB	12	SERPINB7	3	DIO3	1
SPINK1	12	SFRS10	3	DIRAS2	1
TTR	12	SFTPD	3	DLG2	1
VWF	12	SGK	3	DLK1	1
ABCC2	11	SH2D2A	3	DLL3	1
ACP1	11	SKALP	3	DMWD	1
ALDRL2	11	SLC12A6	3	DNAH11	1
APOC1	11	SLC17A1	3	DNM3	1
AURKA	11	SLC17A3	3	DNMT3A	1
BRAF	11	SLC18A1	3	DOCK7	1
CCK	11	SLC22A3	3	DPP10	1
CCKAR	11	SLC22A5	3	DPP4	1
DEFB1	11	SLITRK1	3	DPP6	1
DRD1	11	SMAD6	3	DPT	1
GCK	11	SMARCB1	3	DQ515897	1
HLA-DRB4	11	SOST	3	DRB1	1
HLA-E	11	SOX5	3	DRD1IP	1
HLA-G	11	SOX6	3	DSCAML1	1
HSPA1L	11	SPTBN1	3	DSG1	1
HTR1B	11	ST8SIA6	3	DUSP12	1
HTR2C	11	STCH	3	DYX1C1	1
IGF1R	11	STH	3	EBAG9	1
MAPT	11	SYN2	3	ECGF1	1
MICB	11	SYN3	3	ECHDC1	1

MUC1	11	SYNGR1	3	EDA2R	1
PKD1	11	TAAR6	3	EDG7	1
PTPN2	11	TAC1	3	EDN2	1
SLC11A1	11	TBX5	3	EFEMP1	1
TGFBR1	11	TBXA2R	3	EFHC1	1
TPMT	11	TCN1	3	EFNA5	1
ATG16L1	10	TFAM	3	EHBP1	1
CRHR1	10	TFCP2	3	EHD3	1
CYP3A5	10	TGFB	3	EHF	1
ERBB3	10	TGFBRAP1	3	EHMT1	1
ERCC1	10	TGM5	3	EHMT2	1
ERCC5	10	TIMP3	3	EIF2AK2	1
FGA	10	TIPARP	3	EIF3H	1
FMR1	10	TLR7	3	ELA2	1
GJB2	10	TMEFF2	3	ELMOD2	1
HMGCR	10	TMEM18	3	EN1	1
HSPA1A	10	TNFAIP1	3	ENPEP	1
IL12A	10	TNFSF13B	3	EPHA2	1
KCNQ1	10	TNFSF4	3	EPO	1
LMNA	10	TNMD	3	EPOR	1
MSH2	10	TNXB	3	ER	1
OLR1	10	TOR1A	3	ESPN	1
PDCD1	10	TP73L	3	ETS1	1
PTEN	10	TPO	3	EVI5	1
SELL	10	TRA@	3	EXOC2	1
SFTPA1	10	TRAF1	3	EYA1	1
SH2B3	10	TRB@	3	F10	1
STK11	10	TRHR	3	FABP4	1
TAF1	10	TRIB3	3	FAM113B	1
UCP1	10	TRIM21	3	FAM14B	1
UCP3	10	TSHB	3	FAM5C	1
ABO	9	TTF2	3	FAM98A	1
ADAM33	9	TYK2	3	FBX07	1
AHR	9	TYR	3	FCAR	1
BTNL2	9	UGT1A10	3	FCGR1B	1
CBS	9	VCAM1	3	FDPSP	1
CD36	9	WISP1	3	FEM1A	1
CHRM3	9	WIT1	3	FFAR1	1
CLOCK	9	XBP1	3	FGF20	1
COL3A1	9	XIAP	3	FGL2	1
DISC1	9	XRCC2	3	FIGN	1
FLT3	9	XRCC4	3	FLJ11730	1
GC	9	XYLT1	3	FLJ16641	1
HTR1A	9	ZMAT4	3	FLJ20184	1

IL8RA	9	ZMIZ1	3	FLJ20309	1
KIAA0350	9	ZP1	3	FLJ34870	1
KIT	9	AANAT	2	FLJ42280	1
LIPG	9	ABCA2	2	FNTB	1
LRP1	9	ABCA3	2	FOXE1	1
LTC4S	9	ABCC1	2	FOXL1	1
MTAP	9	ABI2	2	FOXL2	1
NKX2-3	9	ACACA	2	FOXO3	1
P2RX7	9	ACADS	2	FPR1	1
PALB2	9	ACADSB	2	FREQ	1
PDE4D	9	ACHE	2	FRMD6	1
PECAM1	9	ACOT7	2	FRS2	1
PLIN	9	ACSL4	2	G6PC	1
PLXNA2	9	ADAD1	2	G6PC2	1
RB1	9	ADAMTS14	2	GAA	1
RYR1	9	ADAMTS18	2	GAB2	1
SELPLG	9	ADARB1	2	GAK	1
SFTPA2	9	ADD2	2	GALE	1
SLC6A2	9	ADH6	2	GALK1	1
SRD5A2	9	ADORA1	2	GALNACT-2	1
TGFBR3	9	AFF3	2	GALNT13	1
THRB	9	AFP	2	GATA3	1
TNFRSF11B	9	AGC1	2	GCH1	1
UTS2	9	AGTRL1	2	GCLC	1
VKORC1	9	AIRE	2	GDAP1	1
ABCG8	8	ALDH1L1	2	GIPR	1
ADRB1	8	ALDH3A1	2	GJA8	1
ALOX5	8	ALK	2	GJB1	1
BAT1	8	ALOX15	2	GJB6	1
CHRNA4	8	ALPL	2	GLG1	1
CIITA	8	ALPP	2	GMIP	1
COL1A2	8	ALS2	2	GNG4	1
CYP11A1	8	ALX4	2	GNRHR	1
CYP2C8	8	AMAC1L2	2	GOSR2	1
DTNBP1	8	AMACR	2	GP1BB	1
EDNRB	8	ANGPTL4	2	GPC5	1
ELN	8	ANKH	2	GPC6	1
EPHX2	8	ANP32A	2	GPD2	1
FADS2	8	APOBEC3G	2	GPR10	1
FCRL3	8	ARG1	2	GPR126	1
FGFR2	8	ARHGEF11	2	GPR177	1
GABRG2	8	ARHGEF3	2	GPR44	1
GAD1	8	ARMS2	2	GPR50	1
GATA1	8	ARNTL	2	GPX4	1

GRIK1	8	ASF1A	2	GRAMD1B	1
HSD17B1	8	ATIC	2	GRIA3	1
IL10RA	8	ATP1A2	2	GRIA4	1
IL16	8	ATP1B1	2	GRIN3A	1
INSL3	8	ATP7B	2	GSDMA	1
IRGM	8	AVP	2	GSPT2	1
LCAT	8	AVPR2	2	GUCA1A	1
MST1	8	BANK1	2	H19	1
MT-ND2	8	BAT2	2	H6PD	1
NQO2	8	BBS2	2	HAMP	1
PADI4	8	BBS4	2	HbF	1
PCSK9	8	BCL3	2	HBG2	1
PLN	8	BCR	2	HCCA2	1
POLG2	8	BFZB	2	HCK	1
PVT1	8	BHMT	2	HCRT	1
RASSF1	8	BMP2	2	HDAC5	1
RYR2	8	BMP4	2	HEXB	1
SCN1A	8	BRAP	2	HFE2	1
SDHD	8	BTBD9	2	HIC2	1
SHMT1	8	BTLA	2	HLA-DQA2	1
TIMP2	8	BTN1A1	2	HLA-DQB	1
TSHR	8	BYSL	2	HMBS	1
ACSM3	7	C10orf67	2	HOXD13	1
ADRA2B	7	C11orf30	2	HS3ST3A1	1
AGTR2	7	C12orf30	2	HsG57825	1
AKR1B1	7	C13orf31	2	HSPE1	1
ALOX12	7	C17orf53	2	HTR3A	1
APOC2	7	C4BPA	2	HTT	1
BDKRB1	7	C5orf20	2	HUMPONA	1
CHRNA3	7	C6orf12	2	IAPP	1
CHRNA5	7	C7	2	ICOSLG	1
CNTF	7	C8orf13	2	IDUA	1
CYP27B1	7	CALM1	2	IGF2AS	1
ELMO1	7	capon	2	IGHMBP2	1
FCER1A	7	CAPSL	2	IGSF4B	1
FGFR3	7	CAV1	2	IKZF3	1
FOXC2	7	CBLB	2	IKZF4	1
FUT2	7	CCDC100	2	IL11RA	1
GRIN1	7	CCDC101	2	IL13; SLC22A4	1
GSTA1	7	CCKBR	2	IL17F	1
GSTM3	7	CCL1	2	IL1F10	1
HLA-DRA	7	CCL26	2	IL1RAP	1
IRS2	7	CCND3	2	IL22	1
JAG1	7	CCNY	2	IL26	1

KCNH2	7	CCR1	2	IL28A	1
LIG4	7	CD16A	2	IL28B	1
LRP5	7	CD209	2	IL5RA	1
MME	7	CD3E	2	IL7RA	1
MMP12	7	CD40LG	2	IMP5	1
MS4A2	7	CDC2L2	2	INPP4A	1
MSH3	7	CDC42SE2	2	INSL4	1
MYC	7	CDC91L1	2	INSL6	1
NF1	7	CDH13	2	intergenic-HLA	1
NPSR1	7	CDH23	2	INTS12	1
NRAS	7	CDK6	2	IRF5-TNPO3	1
PDGFRA	7	CDKAL	2	IRF8	1
PGC	7	CDRT4	2	ITFG3	1
PSCA	7	CEBPA	2	ITGA6	1
PTGS1	7	CER1	2	ITGA8	1
REN	7	CFHR1	2	ITIH4	1
RPA1	7	CFHR3	2	ITPR2	1
S100B	7	CHAT	2	ITPR3	1
SCNN1B	7	CHDS	2	JMJD1C	1
SHBG	7	CHGB	2	KCNAB1	1
SLC12A3	7	CHRFAM7A	2	KCNE2	1
SLC30A8	7	CHRNB1	2	KCNE4	1
SLCO1B1	7	CIDEA	2	KCNIP1	1
SOD3	7	CLCA1	2	KCNMB2	1
STAT3	7	CLEC4M	2	KIAA0999	1
STAT4	7	CLN3	2	KIAA1267	1
SUMO4	7	CLPS	2	KIAA1727	1
TLR1	7	CNGB3	2	KIAA1841	1
TNFAIP3	7	CNOT1	2	KIF1B	1
TNFRSF10A	7	CNTN4	2	KIF21B	1
TPH2	7	CNTNAP2	2	KIF5A-PIP4K2C	1
UGT1A9	7	COG6	2	KIFAP3	1
USF1	7	COL17A1	2	KIR2DL1	1
WNK4	7	COL4A1	2	KLHL25	1
A2M	6	COMP	2	KLK7	1
ABCB4	6	CPT1B	2	KRTHB5	1
ABCC4	6	CR2	2	LAMB1	1
ADA	6	CRABP2	2	LASS6	1
ADIPOR1	6	CRH	2	LBXCOR1	1
ADM	6	CRHBP	2	LCE3A	1
AKAP10	6	CRYAB	2	LCE3D	1
ALOX5AP	6	CSH1	2	LGI4	1
ATF6	6	CSH2	2	LGR5	1
BCHE	6	CSNK1G2	2	LHFPL2	1

BCL6	6	CST9	2	LILRA3	1
BSN	6	CST9L	2	LINGO1	1
CAPN5	6	CTNNBL1	2	LIPA	1
CART	6	CTSG	2	LMO4	1
CCL11	6	CXCL16	2	LMTK2	1
CELSR2	6	CXCL2	2	LOC122509	1
CFC1	6	CXCR3	2	LOC123688	1
CLEC16A	6	CXCR6	2	LOC132321	1
COL18A1	6	CYBRD1	2	LOC340602	1
CPT1A	6	CYLD	2	LOC344371	1
CTSD	6	CYP27A1	2	LOC399959	1
CYP2A13	6	CYP2R1	2	LOC440069	1
CYP7A1	6	CYP46	2	LOC644246	1
DCTN5	6	CYP46A1	2	LOC645044	1
EDNRA	6	CYP7B1	2	LOC648570	1
F3	6	CYSLTR2	2	LPAL2	1
FADS1	6	DACH2	2	LPIN1	1
GABRA5	6	DAPK1	2	LYPLAL1	1
GABRA6	6	DBP	2	LYZ	1
GABRB2	6	DCK	2	LZTS1	1
GABRB3	6	DDAH1	2	MAF	1
GBA	6	DDC	2	MAGED1	1
GYS1	6	DDX25	2	MAGI2	1
HAPLN1	6	DGCR14	2	MAL2	1
HAVCR1	6	DIDO1	2	MAMDC1	1
Hb	6	DLD	2	MAP2K5	1
HGF	6	DLG4	2	MAP3K7IP2	1
HHEX	6	DLST	2	MAPK1	1
HLA-DPA1	6	DMPK	2	MATN1	1
HPSE	6	DNAH5	2	MATP	1
HSD11B1	6	DNMBP	2	MAX	1
HTR4	6	DOCK3	2	MBTPS2	1
IFNAR1	6	DPCR1	2	MC2R	1
IL15	6	DRIM	2	MCF2L	1
IL24	6	E2F1	2	MCHR1	1
IL2RA	6	EIF3C	2	MCTP2	1
IL5	6	ENDOGL1	2	MDK	1
IMPA2	6	EP300	2	MEG3	1
IRAK1	6	ERAP1	2	MEIS1	1
ITPA	6	ERC2	2	MEPE	1
KIR3DL1	6	EXO1	2	METTL1	1
L1CAM	6	EXOC4	2	MFGE8	1
LCT	6	F9	2	MGC45800	1
LIPE	6	FAM13A	2	MKI67	1

LNK	6	FAM55B	2	MLN	1
MAOB	6	FANCA	2	MMEL1-TNFRSF14	1
MED12	6	FCER2	2	MMRN1	1
MLH3	6	FGF1	2	MOBKL2B	1
MMP7	6	FLJ22536	2	MPHOSPH9	1
MTHFD1	6	FLJ32831	2	MPL	1
MUTYH	6	FLJ44180	2	MRAS	1
MX1	6	FMN1	2	MRPL19	1
MYBPC3	6	FOLH1	2	MRPS6	1
MYLK	6	FOXP3	2	MSMB	1
NDUFAB1	6	FRAXE	2	MT-CO2	1
NEGR1	6	FSCN2	2	MTM1	1
NER	6	FTL	2	MTMR2	1
NLRP3	6	FXN	2	MT-ND5	1
NOD1	6	FZD3	2	MT-TS2	1
NOS1AP	6	GABARAP	2	MUC15	1
NPHS1	6	GABRA2	2	MUC19	1
NR0B2	6	GABRA3	2	MUC2	1
ODC1	6	GAL	2	MUC20	1
P2RY12	6	GALR3	2	MUC3A	1
PARL	6	GAPDH	2	MUC7	1
PDE11A	6	GAPDHS	2	MXI1	1
PDYN	6	GCDH	2	MYADML	1
PKHD1	6	GDF9	2	MYCN	1
PRKCG	6	GDNF	2	MYL2	1
PSEN1	6	GH2	2	MYO15	1
PTGIS	6	GHRH	2	MYOZ2	1
PTPRC	6	GHRHR	2	MYRIP	1
RAD51	6	GHSR	2	NAALADL2	1
SCGB1A1	6	GIMAP5	2	NAPG	1
SFTPC	6	GINS3	2	NCAPD3	1
SNAP25	6	GJA1	2	NDUFA8	1
SNCA	6	GLIS3	2	NEBL	1
SREBF1	6	GLRX	2	NEIL3	1
TCEAL1	6	GNAI2	2	NFATC4	1
TF	6	GNPDA2	2	NGFR	1
TFAP2B	6	GNRH1	2	NHE1	1
TGFB3	6	GOT2	2	NIACR2	1
TGFBR2	6	GP9	2	NINJ2	1
TRAFD1	6	GPR98	2	NKX2	1
VLDLR	6	GREM1	2	NKX2-1	1
ZP4	6	GRIA1	2	NKX3	1
ABCB11	5	GRID1	2	NKX3.1	1
ACE2	5	GRIN2A	2	NLGN4	1

ADH4	5	GRK4	2	NLRP11	1
ADIPOR2	5	GRM3	2	NNMT	1
AGRP	5	GRM8	2	NOLA1	1
AIF1	5	GSDMC	2	NOTCH2	1
APOA	5	GSTCD	2	NP_001013641.2	1
ARL11	5	GSTM4	2	NPAS2	1
ARRB2	5	GSTO2	2	NPC1L1	1
ATP1A1	5	GSTT2	2	NPFFR2	1
BCL11A	5	GTF2A1	2	NPNT	1
C3	5	GUCA1B	2	NPR2	1
CACNA1F	5	H2	2	NRAMP	1
CASP8	5	HABP2	2	NRF1	1
CD19	5	HACE1	2	NRG3	1
CD24	5	HAS1	2	NRIP1	1
CD86	5	HBS1L	2	NRL	1
CEL	5	HCN2	2	NS5A	1
CEP290	5	HERG	2	NSF	1
CHI3L1	5	HES1	2	NT5C1A	1
CLU	5	HESX1	2	NTRK1	1
CST3	5	HEXA	2	NUCKS1	1
CTDSPL	5	HHIP	2	NUDT10	1
CTNNA3	5	HIST1H2BJ	2	NUDT11	1
CYP21A1	5	HLA-D	2	OAT	1
CYP2B6	5	HLA-DMB	2	OBFC1	1
CYP2J2	5	HLA-DR-DQ	2	OPRD1	1
CYP4A11	5	HLA-F	2	OPTC	1
CYP4F2	5	HMGA2	2	OR10J1	1
DAOA	5	HNF1B	2	OR10J3	1
DDAH2	5	HNRPUL1	2	OR10J5	1
DHFR	5	HORMAD2	2	OR13G1	1
DIO2	5	HPN	2	OR2C3	1
DMD	5	HSD17B3	2	OR5AP2	1
ELAC2	5	HSD17B6	2	OR5AR1	1
ENG	5	HSD3B1	2	OR5H2	1
EPHB2	5	HSPA4	2	OR9G1	1
ERCC6	5	HSPA8	2	OR9G4	1
FABP1	5	HSPA9	2	ORF DQ515897	1
FCGR1A	5	HSPG2	2	OTOF	1
FN1	5	HYOU1	2	OTUD3	1
FRAXA	5	ICAM3	2	OVCH2	1
FSHB	5	ICOS	2	P2RX6	1
GABBR1	5	IFNA2	2	P2RY11	1
GABRB1	5	IFNK	2	PAH	1
GAD2	5	IGFBP7	2	PALLD	1

GALT	5	IGLV@	2	PARD3	1
GP6	5	IGLV8	2	PARD3B	1
GRIK3	5	IKBKAP	2	PARK16	1
GRIN2B	5	IKBL	2	PAX1	1
GSTO1	5	IKZF2	2	PAX5	1
HBA1	5	IL11	2	PAX6	1
HCP5	5	IL13RA2	2	PAX8	1
HLA-DMA	5	IL18R1	2	PAX9	1
HLA-DRB3	5	IL18RAP	2	PCDH11X	1
HPV16	5	IL19	2	PCK1	1
HTR6	5	IL1R	2	PDE6B	1
IL12RB1	5	IL20	2	PDE8B	1
IL23A	5	IL6ST	2	PDS	1
IL27	5	IL9	2	PDZK1	1
IL7R	5	INHA	2	PDZRN4	1
IL8RB	5	IPO13	2	PEAR1	1
IRF1	5	IREB2	2	PER2	1
IRF4	5	IRF3	2	PHLDB1	1
KLF6	5	ITGA11	2	PICALM	1
KLK1	5	ITGAX	2	PIK3CG	1
LIG1	5	ITLN1	2	PIK3R1	1
LMAN1	5	JARID2	2	PLA2G1B	1
LOXL1	5	JAZF1	2	PLA2G2A	1
LRRK2	5	KCNA3	2	PLA2G2E	1
MATN3	5	KCNB2	2	PLP1	1
MBD4	5	KCNH8	2	PM20D1	1
MC3R	5	KCNJ2	2	POLB	1
MLC1	5	KCNJ3	2	POLI	1
MLXIPL	5	KIAA0319	2	POLR3B	1
MT2A	5	KIAA1109	2	POU3F4	1
MT-TL1	5	KIAA1542	2	POU5FIP1	1
MYO9B	5	KIF11	2	PPM1E	1
NDUFV2	5	KIR2DL2	2	PPT2	1
NME1	5	KIR2DL5B	2	PRDM1	1
NOS2	5	KIR2DS1	2	PRDM2	1
NOTCH4	5	KLF1	2	PREX1	1
NPHP1	5	KLF7	2	PRKAG2	1
NRG1	5	KLH21	2	PRKCE	1
OAS1	5	KLHL13	2	PRKD2	1
OPTN	5	KRT23	2	PRL	1
PAX2	5	KRT8	2	PRLHR	1
PCSK1	5	LAMA2	2	PRLR	1
PDE3A	5	LDHA	2	PRM3	1
PDX1	5	LEC	2	PRMT8	1

PHOX2B	5	LELP1	2	PROX1	1
PIK3CA	5	LIG3	2	PRPH	1
PITX2	5	LIMK1	2	PRRG4	1
PMCH	5	LOC150577	2	PRSS2	1
PMS1	5	LOC344382	2	PRUNE2	1
PNMT	5	LOC400680	2	PSMG1	1
POLK	5	LOC643714	2	PTCH1	1
POLR2B	5	LOXL2	2	PTER	1
POMC	5	LPP	2	PTGER3	1
PRKCB1	5	LRP4	2	PTGES2	1
PRKCH	5	LRP6	2	PTGIR	1
PROC	5	LRPAP1	2	PTPRD	1
PROCR	5	LRRC16	2	PTPRE	1
PRSS1	5	LTF	2	PVRL2	1
PSMB9	5	LUM	2	PYGM	1
PSRC1	5	LUZP2	2	Q9UD29	1
PTGDS	5	M6PR	2	QKI	1
PTHR1	5	MADD	2	QPCT	1
RPA3	5	MAG	2	QSER1	1
SAA1	5	MAPK8IP1	2	RAB38	1
SAI1	5	MECP2	2	RAB5B	1
SCARB1	5	MEF2C	2	RAB7L1	1
SH2D1A	5	MGC13125	2	RAC2	1
SLC26A4	5	MGST1	2	RAD50	1
SLC2A1	5	MIA3	2	RAD51L1	1
SLC3A1	5	MINK1	2	RAFTLIN	1
SMAD3	5	MIRN146A	2	RAI14	1
SMN1	5	MMP13	2	RANTES	1
TBX21	5	MPZL1	2	RAPGEF3	1
TCN2	5	MSH5	2	RARB	1
TERT	5	MSR1	2	RASA1	1
TFPI	5	MSRA	2	RASGEF1A	1
THBS1	5	MSRB2	2	RASGRP1	1
THBS2	5	MSX1	2	RAX	1
TIMELESS	5	MT3	2	RBMS1	1
TLR10	5	MTCH2	2	RBMX	1
TLR5	5	MTMR3	2	RCL1	1
TRIB1	5	MT-ND3	2	RFK	1
UBL5	5	MTP	2	RFX4	1
UGT1A6	5	MT-RNR1	2	RHPN2	1
UGT2B7	5	MT-TV	2	RNASE1	1
WFS1	5	MUC6	2	RNF146	1
XRCC6	5	MYB	2	RNF186	1
ABCA7	4	NAGS	2	ROCK2	1

ABCC6	4	NCAM1	2	ROM1	1
ADORA2A	4	NCSTN	2	ROS1	1
ADRA1A	4	NDUFS1	2	RP11	1
ANKK1	4	NDUFV3	2	RPGRIP1	1
APOA2	4	NEDD4L	2	RPL21	1
APOM	4	NEFH	2	RPL5	1
AQP7	4	NEFM	2	RPN2	1
ARHGEF10L	4	NET1	2	RS1	1
ATN1	4	NEUROG1	2	RTBDN	1
BAT3	4	NF2	2	RTEL1	1
BCL1	4	NFE2L2	2	RTL1	1
BMP15	4	NID2	2	S100A5	1
BMPR1A	4	NKX3-1	2	SALL3	1
C2	4	NMU	2	SAT	1
C4A	4	NOG	2	SC4MOL	1
c6orf204	4	NOTCH1	2	SCA6	1
C6orf97	4	NPHP4	2	SCAMP1	1
CACNA1A	4	NPM1	2	SCAND1	1
CALCA	4	NPPC	2	SCG3	1
CARD8	4	NPR3	2	SCO2	1
CCR3	4	NPY2R	2	SDC1	1
CD22	4	NRCAM	2	SEMA5A	1
CD70	4	NRP2	2	SEMA6A	1
CDSN	4	NRXN1	2	SERPINA6	1
CHEK1	4	NTF3	2	SERPINE2	1
CHRM1	4	NTNG1	2	SERPING1	1
CHRNA7	4	NTNG2	2	SERPINI1	1
CLCN5	4	NTRK3	2	SETD8	1
CLPTM1L	4	NUBPL	2	SEZ6L2	1
CNDP1	4	NUDT1	2	SFRS2IP	1
COL11A2	4	NUMB	2	SFXN2	1
COX1	4	NUPR1	2	SGK1	1
CPOX	4	NYX	2	SH3GL2	1
CPS1	4	OCA2	2	SIAT7C	1
CRX	4	OLFM2	2	SIRPA	1
CSF2	4	OLIG3	2	SLC01B3	1
CYP21A2	4	OPG	2	SLC14A2	1
CYSLTR1	4	OSX	2	SLC15A4	1
DBI	4	OTX2	2	SLC16A9	1
DCN	4	OXTR	2	SLC22A11	1
DDR1	4	PACRG	2	SLC24A4	1
DDX5	4	PAK1	2	SLC25A36	1
DGKB	4	PALMD	2	SLC26A3	1
DLG5	4	PARD6A	2	SLC2A2	1

DNASE1	4	PARP4	2	SLC39A11	1
DPYD	4	PBX4	2	SLC41A1	1
EPB42	4	PCSK2	2	SLC45A3	1
F11	4	PDE4B	2	SLC5A3	1
FAAH	4	PDGFC	2	SLC6A14	1
FANCC	4	PDLIM5	2	SLC7A1	1
FBN2	4	PECR	2	SLC7A9	1
FBXO11	4	PEMT	2	SLC9A9	1
FCGR2B	4	PER1	2	SLCO3A1	1
FLG	4	PGDS	2	SLIT2	1
FOXP2	4	PHTF1	2	SMAD7	1
FSHR	4	PIG3	2	SOAT2	1
FUT3	4	PIGR	2	SOCS2	1
GAB1	4	PIK3C3	2	SORL1	1
GABRA1	4	PIN1	2	SOX17	1
GATA2	4	PIP5K2A	2	SP110	1
GDF5	4	PKM2	2	SP140	1
GFPT1	4	PLCG1	2	SP7	1
GH1	4	PLD2	2	SPACA1	1
GJA5	4	PLEKHG5	2	SPATA2	1
GLTSCR1	4	PLOD1	2	SPG4	1
GNMT	4	PMP22	2	SPTA1	1
GPX3	4	PNPLA2	2	SRY	1
GSK3B	4	PON3	2	SSTR5	1
HCRTR2	4	POR	2	ST2	1
HMHA1	4	POU1F1	2	ST6GAL1	1
HSD3B2	4	POU2AF1	2	STARD3NL	1
HTR3B	4	POU2F1	2	STAT2	1
HY	4	PPIC	2	STBD1	1
IBD5	4	PPP1R13L	2	STIP1	1
IDE	4	PPP1R1B	2	STK39	1
IGF2BP2	4	PPP1R3	2	STRN4	1
IGF2R	4	PPP2R2B	2	STX1A	1
IGFBP1	4	PPP3CC	2	SUOX	1
IGFBP3	4	PRKCA	2	SUSD1	1
IKZF1	4	PRKCQ	2	SVIL	1
IL13RA1	4	PRKCSH	2	SYBL1	1
IL1RL1	4	PRKCZ	2	SYCP3	1
INSIG2	4	PRND	2	SYNE1	1
IPF1	4	PROZ	2	TAOK1	1
IRF5	4	PSEN2	2	TARBP1	1
KCNE1	4	PSENEN	2	TAS2R16	1
KCNN3	4	pseudogene	2	TAS2R38	1
KDR	4	PSMA4	2	TAS2R50	1

KIR2DS2	4	PSORS1	2	TBCE	1
KLK3	4	PSORS1C1	2	TBX1	1
LMO2	4	PSORS1C3	2	TBXAS1	1
LPA	4	PTAFR	2	TDO2	1
MBOAT5	4	PTGDR	2	TENR	1
MBP	4	PTGER2	2	TERC	1
MERTK	4	PTS	2	TERF1	1
MET	4	PXK	2	TERF2	1
MGP	4	Q4KMW4	2	TES	1
MMP8	4	Q6ZNG6	2	TET2	1
MPZ	4	Q6ZUR9	2	TGFBI	1
MT-ND1	4	RANBP5	2	THBS4	1
MVK	4	RANK	2	THSD4	1
MYO7A	4	RAPGEF5	2	TK2	1
MYOC	4	RASGRP3	2	TLE4	1
NCAN	4	RBMS3	2	TLR8	1
NCOA3	4	RDH12	2	TMCC2	1
NEUROD1	4	REG1A	2	TMEM195	1
NFKBIL1	4	REL	2	TMEM57	1
NMB	4	RENBP	2	TNFIP3	1
NP	4	REV1	2	TNFRSF11A	1
NPHS2	4	RFFL	2	TNFRSF6B	1
NPPB	4	RGS1	2	TNFSF13	1
NPY5R	4	RGS6	2	TNP2	1
NR4A2	4	RHBDD1	2	TNR	1
NR5A1	4	RHD	2	TNRC6B	1
NRXN3	4	RIL	2	TNS1	1
NTRK2	4	RIPK2	2	TOLLIP	1
OLIG2	4	RKHD3	2	TOX3	1
OPA1	4	RNASE3	2	TP63	1
OPRS1	4	RP1	2	TPCN2	1
PADI6	4	RRM1	2	TPK1	1
PAFAH2	4	RSPO3	2	TPM3	1
PAI1	4	RUNX1	2	TPP1	1
PARK2	4	RUNX2	2	TRAM1L1	1
PAX4	4	SA	2	TRH	1
PDCD5	4	SAT1	2	TS13	1
Pending	4	SCGB3A2	2	TSC1	1
PIK3CB	4	SCHIP1	2	TSHZ2	1
PINK1	4	SCN1B	2	TSPAN8	1
PIP	4	SCN2A	2	TWIST1	1
PKLR	4	SCN7A	2	TYRP1	1
PLA2G6	4	SCNN1A	2	UBASH3A	1
PNPLA3	4	SCNN1G	2	UBE2L3	1

POLG	4	SDC3	2	UGT2B17	1
PPARGC1B	4	SEC63	2	UNC13A	1
PRF1	4	selectin	2	USF2	1
PRODH	4	SEPP1	2	USP12	1
PSMB8	4	SERPINH1	2	VPS13C	1
PTH	4	SFN	2	VPS37A	1
PTK2	4	SGCD	2	VSX1	1
PTPRJ	4	SGSM2	2	VTCN1	1
RCC2	4	SIPA1	2	WAS	1
RECQL4	4	SLC10A2	2	WDFY4	1
RELN	4	SLC12A1	2	WDR12	1
RGS4	4	SLC18A2	2	WDR66	1
RHO	4	SLC1A1	2	WDR69	1
RHOU	4	SLC1A2	2	WDR7	1
RNF39	4	SLC25A12	2	XPNPEP2	1
RNR2	4	SLC25A14	2	XRCC5	1
RTN4	4	SLC25A27	2	YEATS4	1
RXRG	4	SLC25A46	2	YWHAE	1
SERPINC1	4	SLC38A7	2	YWHAH	1
SERPINF2	4	SLC40A1	2	ZBP1	1
SH2B1	4	SLC4A1	2	ZFP64	1
SLC22A12	4	SLC4A3	2	ZIC1	1
SLC22A4	4	SLC5A4	2	ZIC2	1
SLC2A9	4	SLC8A1	2	ZNF224	1
SLC35F1	4	SLC9A3R1	2	ZNF230	1
SMAD4	4	SLC9A4	2	ZNF248	1
SORBS1	4	SLIT3	2	ZNF365	1
SORT1	4	SMG6	2	ZNF498	1
SPARC	4	SNAP29	2	ZNF536	1
SPINK5	4	SNN	2	ZNF746	1
SRR	4	SNORD52	2	8-Mar	1
TACR3	4	SNRPN	2		
TCF2	4	SNTG1	2		
TFR2	4	SOAT1	2		
TFRC	4	SOCS6	2		
